# Inequalities in educational outcomes in individuals with childhood experience of out-of-home care: What are driving the differences?

**DOI:** 10.1371/journal.pone.0232061

**Published:** 2020-04-20

**Authors:** Lars Brännström, Hilma Forsman, Bo Vinnerljung, Ylva B. Almquist

**Affiliations:** 1 Department of Social Work, Stockholm University, Stockholm, Stockholm, Sweden; 2 Department of Public Health Sciences, Stockholm University, Stockholm, Sweden; TED University, TURKEY

## Abstract

**Background:**

Prior research has shown that individuals with experience of out-of-home care (foster family care or residential care) in childhood are educationally disadvantaged compared to their peers. In order to be better equipped to design interventions aimed at improving the educational outcomes of children for whom society has assumed responsibility, this study seeks to further our understanding about which factors that contribute to the educational disparities throughout the life course.

**Methods:**

Using longitudinal data from a cohort of more than 13,000 Swedes, of which around 7% have childhood experience of out-of-home care, Peters-Belson decomposition is utilized to quantify the extent to which the gap in educational achievement in school (age 16) and midlife educational attainment (age 50) captures differences in the prevalence of factors influencing educational outcomes, and differences in the impacts between these factors.

**Results:**

We find that the achievement and the attainment gap was around 13% and 9% respectively. These gaps were to a large extent explained by differences in the distribution of predictors. The major explanatory factor for placed children’s lower achievement was a lower average cognitive ability. Yet there were some evidence that the rewards of cognitive ability in these children differed across the life course. While the lower returns of cognitive ability suggest that they were underperforming in compulsory school, the higher returns of cognitive ability on midlife attainment indicate that–given previous underperformance–their attainment at age 50 reflects their cognitive capacity more accurately than their achievement at age 16 do.

**Conclusion:**

The large influence of the unequal distribution of predictors suggests that policy efforts are needed to promote equity in the distribution of factors contributing to educational achievement and attainment. Since cognitive ability was found to be an important contributory factor, such efforts may include promoting cognitive and intellectual development among children in out-of-home care, preferably starting at a young age.

## Introduction

Empirical research from all over the Western world has for decades reported that individuals with a childhood history of being placed in out-of-home care (OHC; foster family or residential care) perform very poorly in the educational system [1–4]. Research on child maltreatment, which is not restricted to children with experience of placement in OHC, has also amassed substantial evidence demonstrating that experiences of child abuse and neglect are associated with poor educational outcomes [5, 6].

The empirical links between poor educational achievement/attainment and unfavourable later-life outcomes are strong among children in both general [7] and vulnerable populations such as child welfare clients [8]. Although these links do not necessarily reflect causal relations, the association between poor educational achievement and adverse developmental outcomes in individuals with experience of OHC seems to allow for causal interpretations [9]. Since educational success has been linked to better life-course outcomes [10], improving educational outcomes in OHC populations seems to represent a viable strategy to prevent negative development [11–13]. However, to be better equipped to design interventions aimed at improving the educational outcomes of children for whom society has assumed responsibility, we need to further our understanding about which factors that contribute to the educational disparities [14].

Using longitudinal data from a cohort of more than 13,000 individuals born in 1953 and living in Metropolitan Stockholm (the capital region of Sweden) at age 10, of which around 7% have had experience of OHC during their upbringing, this study aims to decompose inequalities in educational outcomes between individuals with and without a childhood history of placement in OHC into its contributory factors. This is achieved by utilizing Peters-Belson (PB) decomposition [15, 16] which allows us to quantify the extent to which the gap in educational achievement and attainment between OHC and non-OHC groups throughout the life course reflects differences in the prevalence of factors influencing educational outcomes, and differences in the impacts between these factors.

Our analyses extend prior research in several ways. First, and in contrast to many previous studies, we avoid inherent shortcomings of cross-sectional data and we minimize retrospective bias related to self-reports or parental reports about experiences of OHC. Our birth cohort sample also includes a large number of individuals with OHC experience. Second, we have unusually rich data on cohort member’s biological parental socio-economic circumstances, variable risk factors such as the individual’s scholastic ambitions, future orientations, behavioural problems, cognitive skills, and reasons for placement, all of which have been hypothesized to explain the educational gap between OHC and non-OHC populations [14, 17]. Third, the longitudinal design renders it possible to examining educational attainment in midlife. This is important since free adult education is well developed in Sweden and contributes to the educational system to be inclusive over the life course. Finally, distinguishing between explanations referring to differences in the distribution of determinants of educational outcomes on the one hand, and differences in the impacts of these determinants on the other, is important for policymakers and professionals in child welfare and educational settings since this implies different solutions.

If individuals with and without OHC experience enter the educational system with different needs (i.e. differences in the distribution of determinants of educational outcomes), then we would not expect them to have the same experiences in that system. Put differently, we know that individuals with adverse rearing backgrounds are disproportionally distributed between OHC and non-OHC groups, which suggests that the observed educational disparities are due to selection processes. In such a situation, policy efforts aiming to improve educational outcomes in the OHC group need to target the unequal distribution of factors influencing educational outcomes. However, if two otherwise similar individuals with and without OHC experience enter the educational system and have different experiences (i.e. differences in the impacts between determinants of educational outcomes), we have to delve deeper into the differences in the way the system influences each group.

## Material and methods

### Study population

Longitudinal data from the Stockholm Birth Cohort study (SBC), defined as all individuals born in 1953, who were living in the greater Stockholm metropolitan area ten years later, and were alive and resident in Sweden in 1980 and/or 1990 were used [18]. The SBC was created by a probability matching of two datasets: The Stockholm Metropolitan study (SMS), and The Swedish Work and Mortality Database (WMD). The SBC includes survey and administrative register data from birth for 14,294 individuals (7,305 men, 6,989 women), of which around 9% had experience of OHC at some point between birth and age 19 (1953–1972). For the purposes of this study, the study population consists of children who participated in one of the surveys included in the SMS (the 1966 School Study) and who were alive at age 50 and resident in Sweden. Mortality and migration reduced the sample by around 6%, leaving us with a sample of 13,425 individuals.

To reduce problems related to sample heterogeneity in OHC populations, cohort members who had their first placement as teenagers (n = 268) were excluded from the analytical sample. This strategy further reduced the sample to 13,157 individuals of which 6.8% (n = 896; 474 men, 422 women) primarily had been placed in OHC due to family circumstances (abuse, neglect, parental substance abuse or mental ill health etc.). The OHC group mainly consists of children with short placements (<2 years), and most of them were placed in early childhood (age 0–6 years).

The original study (SMS) was established at a time when informed consent was generally not a part of research practice. For the 1966 School Study, for example, consent from cohort member’s parents or guardians was not necessary. It was sufficient to obtain consent from the education authorities, teachers’ organisations, and the national parents’ and school association [19]. Despite this, the Stockholm regional ethics committee later gave their permission to include these data in the SBC [20]. Ethical permission for the current study was obtained from the Stockholm Regional Ethics Committee (no 2016/481-31/5).

Our cohort members were born at a time when the Swedish welfare state was rapidly expanding and living conditions were improving. Their journey through the educational system was accompanied by the implementation of nation-wide reforms which involved, among other things, an extension of the amount of schooling as well as changes in curricula. Although the educational system became increasingly egalitarian, there was still some tracking for the 1953 cohort. For example, in grade 6, students had to choose between electives, such as more or less advanced English and mathematics. This subsequently led to students being divided into upper secondary preparatory classes and vocational preparatory classes in grade 9. Of relevance for cohort members was also the introduction of a tuition-free adult educational system in 1968, which enabled individuals with unfinished compulsory or upper secondary schooling to complement their education.

It is reasonable to assume that the children who entered OHC in the 1950s and 1960s differ in some regards when compared to the OHC population of today. For the 1953 cohort, frequently occurring reasons for placement were child abuse and neglect, alcohol abuse, parental mental health problems, presumed maternal immaturity (teenage mothers), poor housing conditions, and poverty. Some children were also placed by request from single mothers who were ill, or who lacked sufficient support networks or financial means. Today, it is more common for children to be placed due to child abuse, maternal drug abuse, and domestic violence. On the one hand, this may imply that concurrent cohorts of children in OHC have had more adverse experiences (e.g. traumas) before entering care compared to earlier cohorts. On the other hand, national inquiries into foster and residential care suggest that cases of inferior and hostile care were more common in earlier cohorts [21].

### Variables

Educational outcomes are indicated by two variables: educational achievement in the ninth and final year of compulsory school (1969, age 16), and midlife educational attainment (2003, age 50) (Table 1). Ranging from 100 to 500, educational achievement in ninth year consists of mean grades in the final year of compulsory school (school-leaving certificate). These grades were constructed as to have a Gaussian distribution at the national level with a mean value of approximately 300. Based on the highest out of a seven levels of education (ranging from incomplete compulsory education to post-graduate education) achieved at age 50, the level of educational attainment has been assigned a value corresponding to the number of years typically related to have passed each level (thus reflecting pseudo-years of education). For example, having completed the nine-year compulsory school yields nine pseudo-years. In a similar way, the completion of two years vocational schooling or three years upper secondary schooling means 11 and 12 pseudo-years respectively.

**Table 1:** Sample properties: Descriptive statistics.

Variable	n	Min-max	Proportion/ mean (std. dev.)	% missing
Any placement in OHC (ages 0–19)	13,153	0–1	0.068	-
Educational achievement 9^th^ grade, mean grades (age 16)	11,967	100–500	318.55 (76.46)	9.0
Midlife educational attainment, pseudo-years (age 50)	13,153	6–19	12.33 (2.30)	-
Female	13,153	0–1	0.494	-
Special education, 6^th^ grade (age 13)	13,153	0–1	0.046	-
Cognitive ability 6^th^ grade, test scores (age 13)	11,819	12–116	68.35 (17.90)	10.1
Feelings of safety in school, 6^th^ grade (age 13)	11,776	0–10	6.38 (2.34)	10.5
Interest in schoolwork, 6^th^ grade (age 13)	11,775	0–10	5.01 (2.47)	10.5
Disruptive behavior in school, 6^th^ grade (age 13)	11,767	0–1	0.128	10.2
Future orientation, 6^th^ grade (age 13)	11,613	1–5	3.10 (0.64)	11.7
Self-regulatory skills, 6^th^ grade (age 13)	11,766			10.2
Certainly 100 SEK now		0–1	0.06	
Probably 100 SEK now		0–1	0.07	
Cannot choose		0–1	0.09	
Probably 1000 SEK in five years		0–1	0.35	
Certainly 1000 SEK in five years		0–1	0.43	
Parental attitude towards education, 6^th^ grade (age 13)	10,951	0–10	6.02 (2.32)	16.7
Household poverty, years (ages 0–6)	13,153	0–7	0.39 (1.22)	-
Household occupational class (age 0)	12,747			3.1
Working class, unskilled workers		0–1	0.19	
Working class, skilled workers		0–1	0.29	
Lower middle class, entrepreneur		0–1	0.06	
Lower middle class, officials and non-agricultural employees		0–1	0.32	
Upper and upper middle class		0–1	0.14	
Unmarried mother (age 0)	13,153	0–1	0.114	-
Complete cases	9,792			Total 25.6

OHC = Out-of-home care. SEK = Swedish currency (kronor).

A number of variables associated with educational outcomes in individuals with and without childhood experiences of OHC were used (Table 1). If not otherwise stated, these independent variables refer to circumstances in the sixth and final year of intermediate level of compulsory school (1966, age 13).

Special education refers to a binary variable taking the value of one if the cohort member attended a special class that addressed individual differences and needs. The total number of points on a then commonly used spatial, verbal and numeric test indicates cognitive ability (Härnqvist, 1968). Feelings of safety in school is measured by the sum of 10 two-response items (yes = 1, no = 0) addressing various aspects of safety in school and in the classroom. In a similar way, the total points of 10 two-response items on scholastic ambitions indicates interest in schoolwork. Disruptive behaviour in school was measured by a binary variable taking the value of one if the cohort member reports that (s)he has been told to leave the classroom more than twice. Although this variable may be sensitive to teaching bias towards OHC students, data do not include broader measures of socio-emotional outcomes.

Children’s future orientation is measured using the following question: “If you compare your future prospects with those of your age, do you think your future will be worse, similar or better? Five response alternatives were given: 1. Much worse, 2. A little worse, 3. Just as good, 4. A little better, and 5. Much better. The ordinal variable is treated as continuous in the analyses. Self-regulatory skills are a measure of the capacity to suppress immediate gratification for the benefit of long-term reward and was measured by the following five-response question: “If you had to choose between SEK 100 now or SEK 1000 in five years, which would you choose?” were used. The given response alternatives were 1. Certainly 100 SEK now, 2. Probably 100 SEK now, 3. Cannot choose. 4. Probably 1000 SEK in five years, 5. Certainly 1000 SEK in five years. In today’s worth (2019), SEK 100/1000 refer to around USD 98/980. The variable is treated as categorical in the analyses. Although this variable to some extent may reflect income scarcity, we lack additional measures capturing related personal attributes. Biological parental attitude towards education was measured using the total points of 10 two-response items (yes = 1, no = 0) addressing the child’s experiences of his/her parent’s attitude towards various aspects of schooling (e.g. ‘Do your parents think that having a higher education will give a more secure future?; ‘Do your father and mother consider that lack of education is a serious handicap if one wants to get on in life?’).

Household poverty (1953–1959, ages 0–6) is measured by the number of years the cohort member’s birth family received means-tested social assistance. Based on a then established classification scheme [22], household occupational class in 1953 (age 0) reflects the position of the head of the household (typically the biological father), where higher values generally indicate higher occupational prestige (1. Working class, unskilled worker; 2. Working class, skilled worker; 3. Lower middle class, entrepreneur; 4. Lower middle class, officials and non-agricultural employees; 5. Upper and upper middle class). Due to unclear hierarchy between the third and fourth categories, however, the variable is treated as categorical in the analyses. Unmarried mother (age 0) is a binary variable taking the value of one if the biological mother was unmarried. In the 1950s, being an unmarried woman with children is a proxy for single mother. Sex is a binary variable taking the value of one if the cohort member is female (age 0).

### Statistical analyses

Simple descriptive analysis of the sample and variables are presented in Table 1. Bivariate comparisons of the OHC and non-OHC group with results from two-sample tests of proportions/means are reported in Table 2. While data on midlife educational attainment and variables for socio-demographics such as household poverty are available for all individuals in the analytical sample, other variables have missing values for around 3% to 17% of the observations (Table 1). Using complete cases only, however, yielded an effective sample of 9,972 individuals, a reduction of around 26%. Incomplete data were more frequent in the OHC group (41.1% of the observations) compared to the non-OHC group (24.4% of the observations) (not shown in table). To address the issue of incomplete data, multiple imputation (MI) with full conditional specification [23] were used.

**Table 2:** Group-specific descriptive statistics: Proportions/means (standard deviations within parentheses).

Variable	n	OHC	n	Non-OHC	Difference
Educational achievement 9^th^ grade, mean grades (age 16)	703	288.08 (74.07)	11,264	320.43 (76.22)	-32.35***
Midlife educational attainment, pseudo-years (age 50)	896	11.36 (2.19)	12,257	12.40 (2.29)	-1.04***
Female	896	0.471	12,257	0.496	-0.025
Special education, 6^th^ grade (age 13)	896	0.126	12,257	0.040	0.086***
Cognitive ability 6^th^ grade, test scores (age 13)	734	59.68 (18.76)	11,085	68.92 (17.69)	-9.24***
Feelings of safety in school, 6^th^ grade (age 13)	728	5.82 (2.38)	11,048	6.42 (2.34)	-0.60***
Interest in schoolwork, 6^th^ grade (age 13)	731	5.01 (2.41)	11,044	5.01 (2.48)	0.00
Disruptive behavior in school, 6^th^ grade (age 13)	725	0.162	11,042	0.126	0.036**
Future orientation, 6^th^ grade (age 13)	720	3.06 (0.73)	10,893	3.10 (0.63)	-0.04
Self-regulatory skills, 6^th^ grade (age 13)	726		11,040		
Certainly 100 SEK now		0.077		0.060	0.017
Probably 100 SEK now		0.081		0.066	0.015
Cannot choose		0.107		0.091	0.016
Probably 1000 SEK now		0.338		0.357	-0.019
Certainly 1000 SEK now		0.397		0.423	-0.026
Parental attitude towards education, 6^th^ grade (age 13)	661	5.34 (2.38)	10,290	6.06 (2.31)	-0.72***
Household poverty, years (ages 0–6)	896	1.85 (2.32)	12,257	0.28 (1.01)	1.57***
Household occupational class (age 0)	859		11,888		
Working class, unskilled workers		0.297		0.186	0.111***
Working class, skilled workers		0.378		0.279	0.099***
Lower middle class, entrepreneur		0.069		0.063	0.006
Lower middle class, officials and non-agricultural employees		0.228		0.326	-0.098***
Upper and upper middle class		0.028		0.145	-0.117***
Unmarried mother (age 0)	896	0.315	12,257	0.099	0.216***

OHC = Out-of-home care. SEK = Swedish currency (kronor).

***/**/* indicates statistical significance at the 0.1/1/5% level respectively

Based on 20 imputed datasets, we applied PB decomposition to explain educational disparities between the OHC and non-OHC group. PB decomposition–in economics known as Blinder-Oaxaca decomposition [24, 25]–is a commonly used regression-based technique to explain the difference in the means of an outcome between two groups by decomposing it into two parts. The first (explained) part consists of the difference attributable to between-group differences in the predictors of the outcome, i.e. differences in endowments. The second (unexplained) part comprises differences in the impact that these predictors have on the outcome, i.e. differences in returns to those endowments. In the current study, the first part thus reflects the mean increase in the OHC group’s outcome if they have had the same distribution (i.e. means) of observed characteristics as the non-OHC group. The second part reflects in turn the change in the OHC group’s outcome when applying the non-OHC group’s regression coefficients (slopes) to the OHC group’s characteristics.

The decomposition analysis is performed as follows. First, separate linear regression (ordinary least squares/OLS) models were estimated for the OHC group and non-OHC group to examine group differences in predictors of educational outcomes. Second, PB decomposition regression models are estimated to quantify the gap in the addressed educational outcomes and to identify characteristics contributing to the disparity. For ease of exposition, the contribution of the variables self-regulatory skills and household occupational class, whose impacts are estimated by means of dummy variables reflecting response alternatives, is summed up in a coefficient representing each variable. All analyses were performed using Stata 15/MP-version. The MI module and related commands were used to impute missing data [26]. The PB decompositions were estimated by means of the user written oaxaca-command [27]. To allow for interpreting the educational gap in terms of percent, the natural logarithm of the dependent variables was used.

## Results

### Descriptive statistics

While Table 1 shows descriptive statistics for the total analytical sample, Table 2 presents descriptives for OHC and non-OHC groups respectively. With few exceptions, OHC children tended to have significantly (p<0.01) poorer levels of the addressed variables compared to their same-aged peers without OHC experience. While OHC children had significantly lower values with respect to educational outcomes, cognitive ability, feelings of safety in school, and parental attitude towards education, they had significantly higher values of the degree of having experienced household poverty. They also had higher prevalence rates with respect to special education, disruptive behaviour in school, lower household occupational class, and having an unmarried biological mother at birth. However, there were no statistically significant differences between the two groups regarding the distribution of the sexes, levels of self-regulatory skills, interest in schoolwork, and future orientation.

### Group-specific regression estimates

The left half of Table 3 presents group-specific OLS estimates from a multiple regression analysis where educational achievement in ninth grade is the dependent variable. For the OHC group, all determinants had the expected direction and five out of 12 (41%) were statistically significant. Higher values of cognitive ability and parental attitude towards education were significantly associated with higher grades in the final year of compulsory school. Girls also had higher grades than boys. Disruptive behaviour and household poverty were significantly associated with lower achievement. For the non-OHC group, most of the estimated associations also had the expected direction and were more predictive, at least in the sense that nearly all of the impacts were statistically significant. The latter is perhaps not surprising given the large number of observations (n>12,000).

**Table 3:** Group-specific OLS regression estimates (standard errors within parentheses), by dependent variable.

Variable\Outcome	Educational achievement 9^th^ grade (age 16)		Midlife educational attainment (age 50)	
	OHC	Non-OHC	OHC	Non-OHC
	b-coefficient	b-coefficient	b-coefficient	b-coefficient
Educational achievement 9^th^ grade (age 16)	-	-	0.001*** (0.000)	0.001*** (0.000)
Female	0.042* (0.020)	0.043*** (0.004)	0.054*** (0.013)	0.025*** (0.003)
Special education (age 13)	0.002 (0.032)	0.019 (0.011)	-0.006 (0.020)	-0.047*** (0.007)
Cognitive ability (age 13)	0.005*** (0.001)	0.006*** (0.000)	0.002*** (0.000)	0.001*** (0.000)
Feelings of safety in school (age 13)	0.006 (0.004)	0.011*** (0.001)	0.003 (0.003)	0.001 (0.001)
Interest in school-work (age 13)	0.006 (0.004)	0.007*** (0.001)	-0.001 (0.003)	0.000 (0.001)
Disruptive behavior in school (age 13)	-0.073* (0.030)	-0.058*** (0.007)	0.014 (0.020)	0.011* (0.005)
Future orientation (age 13)	0.007 (0.015)	0.026*** (0.004)	0.009 (0.009)	0.010*** (0.002)
Self-regulatory skills (age 13)				
Probably 100 SEK now	0.025 (0.046)	0.035** (0.012)	-0.002 (0.032)	0.005 (0.008)
Cannot choose	0.021 (0.047)	0.031** (0.011)	-0.011 (0.031)	0.007 (0.008)
Probably 1000 SEK in five years	0.016 (0.041)	0.035*** (0.009)	-0.000 (0.024)	0.007 (0.006)
Certainly 1000 SEK in five years	0.026 (0.039	0.032** (0.009)	0.013 (0.025)	0.007 (0.006)
Parental attitude towards education (age 13)	0.014** (0.005)	0.009** (0.001)	0.013*** (0.003)	0.012*** (0.001)
Household poverty (ages 0–6)	-0.013** (0.004)	-0.007** (0.002)	0.001 (0.003)	-0.004** (0.001)
Household occupational class (age 0)				
Working class, skilled worker	0.000 (0.023)	0.013* (0.006)	0.021 (0.015)	0.013** (0.004)
Lower middle class, entrepreneur	0.000 (0.041)	0.033*** (0.009)	0.067** (0.025)	0.036*** (0.006)
Lower middles class, officials and non-agricultural employees	0.034 (0.028)	0.036*** (0.006)	0.044** (0.017)	0.043*** (0.004)
Upper and upper middle class	-0.001 (0.061)	0.093*** (0.008)	0.053 (0.036)	0.083*** (0.005)
Unmarried mother (age 0)	-0.011 (0.020)	-0.030*** (0.007)	-0.010 (0.012)	-0.020*** (0.005)

Multiple imputation estimates. Outcome variables are measured on the logarithmic scale. OLS = Ordinary least squares. OHC = Out-of-home care.

***/**/* indicates statistical significance at the 0.1/1/5% level respectively. Intercepts suppressed.

Corresponding estimates from a model where midlife educational attainment is the outcome are reported in the right half part of Table 3. Higher values of educational achievement in ninth grade, cognitive ability, parental attitude towards education, and household occupational status were significantly associated with more years of education in both groups. Females also had significantly more years of education compared to males. In addition to the above, having a more positive view of one’s future prospects was also found to be significantly associated with more years of education in the non-OHC group. Unexpectedly, it was also found that experience of special education was associated with more years of education. For the non-OHC group, household poverty, and having an unmarried mother at birth were significantly associated with less years of education.

### Decomposition estimates

Based on the regression estimates reported above, Table 4 depicts the primary results of the decomposition models. Reported in the left-hand part of the table, the decomposition partitioned the 0.125 difference in educational achievement into the difference explained by the means (0.105) and the difference explained by the slopes (0.020). The difference in achievement suggests that the OHC group had approximately 13% lower grades than the non-OHC group, and the gap was largely explained by differences in the distribution of predictors (0.105/0.125 = 84%). In the right-hand part of the table, corresponding estimate for the difference in midlife educational attainment is 9% (0.089). Again, this gap was largely explained by differences in the distribution of predictors (0.079/0.089 = 89%). The estimates of the explained part (0.105 and 0.079) suggest that the OHC group would have increased their grades and years of education with approximately 11% and 8% respectively if they have had the same distribution of observed predictors as the non-OHC group.

**Table 4:** Educational disparities between individuals with and without OHC experience: Overall Peters-Belson decomposition (standard errors within parentheses), by dependent variable.

	Educational achievement 9^th^ grade (age 16)	Midlife educational attainment (age 50)

Difference/Gap ^a)^	0.125*** (0.011)	0.089*** (0.007)
Means/Explained ^b)^	0.105*** (0.007)	0.079*** (0.005)
Slopes/Unexplained ^c)^	0.020* (0.010)	0.010 (0.006)

Multiple imputation estimates (n = 13,153).

^a)^ Refers to the mean difference in predicted (log) outcome between the two groups (based on the regression models reported in Table 3).

^b)^ Part of the outcome differential that is explained by group differences in the predictors. It quantifies the mean increase in the outcome if the OHC group had the same characteristics as the non-OHC group.

^c)^ Part of the outcome differential that is not explained by group differences in the predictors. It quantifies the change in the OHC group when applying the non-OHC group’s coefficients to the OHC characteristics.

***/**/* indicates statistical significance at the 0.1/1/5% level.

The unexplained part in the lower half of the table reflects the change in the OHC group’s outcome when applying the non-OHC group’s regression coefficients (slopes) to the OHC group’s characteristics and suggests that in such a scenario the OHC group will improve their grades and years of education with 2% and 1% respectively (0.020 and 0.010). However, the unexplained part for midlife educational attainment is not statistically significant (p>0.1).

Table 5 provides detailed information about which determinants contribute most to the observed disparities reported above. As shown in the left-hand part of the table, it becomes clear that cognitive ability contributes most to the achievement gap in ninth grade (0.057). Other measures of individual and variable factors that significantly contribute to the disparity are feelings of safety at school (0.006), disruptive behaviour (0.002), and parental attitude towards education (0.007). However, socioeconomic conditions, here indicated by household poverty (0.013), household occupational class at birth (0.012), and having an unmarried mother at birth (0.006) also contribute to the achievement gap. When summarizing all of these estimates, they account for 98% of the explained achievement gap (0.103/0.105 = 0.98). Corresponding significant contributing factors for the midlife attainment gap are educational achievement in ninth grade (0.033), special education (0.004), cognitive ability (0.012), and parental attitude towards education (0.010), as well as household poverty (0.004), household occupational class (0.012), and unmarried mother (0.004). Taken together, these factors accounted for all of the explained attainment gap (0.079/0.079 = 1).

**Table 5:** Contributing factors for educational disparities between individuals with and without OHC experience: Detailed Peters-Belson decomposition (standard errors within parentheses), by dependent variable.

Variable\Outcome	Educational achievement 9^th^ grade (age 16)		Midlife educational attainment (age 50)	
	Means/Explained	Slopes/Unexplained	Means/Explained	Slopes/Unexplained
	Contribution	Contribution	Contribution	Contribution
Educational achievement 9^th^ grade (age 16)	-	-	0.033*** (0.003)	0.071** (0.031)
Female	0.001 (0.001)	0.010 (0.010)	0.001 (0.001)	-0.014* (0.006)
Special education (age 13)	-0.001 (0.001)	0.002 (0.004)	0.004*** (0.001)	-0.004 (0.002)
Cognitive ability (age 13)	0.057*** (0.004)	0.100** (0.037)	0.012*** (0.001)	-0.068** (0.026)
Feelings of safety in school (age 13)	0.006*** (0.001)	0.025 (0.027)	0.001 (0.001)	-0.012 (0.018)
Interest in school-work (age 13)	0.000 (0.000)	0.004 (0.023)	-0.000 (0.000)	0.007 (0.014)
Disruptive behavior in school (age 13)	0.002** (0.001)	0.002 (0.005)	-0.000 (0.000)	-0.001 (0.003)
Future orientation (age 13)	0.001 (0.001)	0.059 (0.050)	0.000 (0.000)	0.002 (0.029)
Self-regulatory skills (age 13) ^a)^	0.001 (0.001)	-0.001 (0.035)	0.000 (0.000)	-0.013 (0.022)
Parental attitude towards education (age 13)	0.007*** (0.001)	-0.025 (0.026)	0.010*** (0.001)	-0.004 (0.017)
Household poverty (ages 0–6)	0.013*** (0.003)	0.011 (0.007)	0.004* (0.002)	-0.006 (0.004)
Household occupational class (age 0) ^a)^	0.012*** (0.001)	0.023 (0.019)	0.012*** (0.001)	0.007 (0.011)
Unmarried mother (age 0)	0.006*** (0.002)	-0.006 (0.006)	0.004*** (0.001)	-0.003 (0.004)

Multiple imputation estimates (n = 13,153). Outcome variables are measured on the logarithmic scale. OHC = Out-of-home care.

^a)^ The sum of the dummy variables contribution.

***/**/* indicates statistical significance at the 0.1/1/5% level respectively. Intercepts suppressed.

When applying the slopes from the non-OHC group to the OHC group’s characteristics, there was a statistically significant impact of cognitive ability on the achievement gap (0.097, p<0.01). The positive estimate suggests that the OHC group will be favoured with respect to educational achievement if their coefficient for cognitive ability was substituted with the non-OHC group’s coefficient. Focusing on the attainment gap, there was a statistically significant impact of the coefficient for educational achievement in ninth grade (0.071, p<0.05), females (-0.014, p<0.05) and cognitive ability (-0.068, p<0.05). The positive coefficient of educational achievement indicates that individuals with OHC experience will benefit if the corresponding coefficient for the non-OHC group was applied to the characteristics for the OHC group. The negative estimates suggest that females with OHC experience actually will be disfavoured with respect to midlife educational attainment if the coefficient for females was substituted with the non-OHC group’s corresponding coefficient. This pattern is also valid for the coefficient for cognitive ability.

## Discussion

Individuals with OHC experience are known to be educationally disadvantaged compared to majority population peers. Prior research has identified a number of characteristics that are associated with educational achievement and attainment in both majority and vulnerable populations such as child welfare clients, and many of these factors are known to be disproportionally found among individuals with a childhood history of placement in OHC. Yet the educational disparities between individuals with and without OHC experience are not sufficiently understood. The current study addressed this issue empirically by using PB decomposition to differentiate between group differences in endowments and in returns to those endowments.

Our results indicate that the patterns of predictors of educational achievement in the ninth and final year of compulsory school and midlife educational attainment to some extent differ between the OHC and non-OHC groups. When decomposing the observed gaps in educational achievement and attainment, our results specify that the disparities almost exclusively could be attributed to differences in endowments, i.e. differences in the distribution of predictors between the two groups. Looking at different types of predictors, we can see that the sum of all individual measures contribute more than the sum of all measures of socio-economic factors. This finding could be of particular policy and practice interest since the individual measures represent variable risk factors. More specifically, lower cognitive ability in the OHC group was the main driving factor behind the achievement gap, while lower average grades contributed most to the midlife attainment gap. Other factors of long-term importance, and which are also theoretically possible to influence through interventions, were lower cognitive ability and lower level of parental attitudes towards education.

Furthermore, there was some evidence that the returns of cognitive ability, i.e. when the slope for cognitive ability in the non-OHC group was applied to the OHC group’s characteristics, actually may favour educational achievement in ninth grade but would disfavour midlife educational attainment in OHC individuals. The rewards of cognitive ability as measured by school performance in the ninth grade were thus lower for OHC-children compared to same-aged peers and higher as measured by educational attainment in midlife. There was also some evidence indicating that the rewards of educational achievement at age 16 on midlife educational attainment were lower in the OHC population.

How to understand these differences in returns of cognitive ability and achievement? The lower returns of cognitive ability suggest that they were underperforming in school, which has previously been noted in Swedish national population studies of cohorts born 1973–82 [28]. Yet, the higher returns of cognitive ability on midlife attainment indicate that–given previous underperformance–their attainment at age 50 reflects their cognitive capacity more accurately than their achievement at age 16 do. In terms of the cognitive ability reward on educational outcomes, this suggest that children with OHC experience are catching up with their same-aged peers without such an experience. The lower returns of achievement on attainment may indicate that OHC children have lower attainment compared to same-aged peers with the same level of achievement. In other words, OHC children are less likely to continue to further education, even when their previous performance allows them [29].

The major explanatory factor for OHC-children’s lower grades was a lower average cognitive ability compared to non-OHC peers. Cognitive and intellectual development is influenced by several factors, genetically and environmentally related. Examples of the latter are familial poverty and early childhood deprivation. However, French adoption studies with sibling-comparison design have shown that cognitive ability among children from adverse backgrounds can be substantially enhanced through improved rearing conditions [30, 31]. Also, intervention studies with pre-post design targeting school performance of children in foster care have reported significantly improved results over time on cognitive tests [32, 33]. In other words, OHC-children’s scores on IQ-tests should not be seen solely as a static trait but rather as a vulnerability that–at least to a certain degree–could be reduced by interventions. If such interventions were successful, the results in this study suggest that this could improve school achievements among OHC-children but less so their educational attainment in a life-course perspective, at least for the birth cohort that has been analysed here.

Strengths of this study include its prospective design with a 50-year follow-up time (as far as we know, longer than any previous prospective study addressing educational outcomes in a child welfare population), the large sample size which included a large number of individuals with OHC experience, data on scholastic ambitions, future orientations, behavioural problems, and socio-economic circumstances of the biological parents. Set against these strengths, a number of limitations should be considered. The policy and practice differences between the child welfare system when the present sample was placed in OHC and today limit the generalizability of the present findings. In Sweden, as in other western countries, there has been a movement toward professionalized care. In addition, there have also been changes in the educational system. Reduced tracking in the compulsory school and the introduction of a voucher system further limit the generalizability. Our indicators of disruptive behaviour and self-regulatory skills may moreover introduce some bias. We also lack data on children’s OHC experiences. Knowing to what extent cohort members had experienced high- or low-quality care, and stable or unstable placement (including potentially involuntary school changes), would probably have promoted our understanding of the educational disparity between OHC and non-OHC groups. As consequence of this unobserved confounding, our estimates may not allow for causal interpretation. Yet, the educational disparities reported in the literature seem to be stable across time and geography [4]. Results from the current study may therefore yield insights into generic processes that shape educational pathways.

At least two implications may be drawn from this study. First, PB decomposition is a useful tool that could be more widely used to further our understanding about educational disparities between individuals with and without experiences of OHC. Rather than simply documenting that OHC populations are educationally disadvantaged, future research should seek to explain this disadvantage. The PB decomposition approach seems like a viable tool here since it identifies and quantifies specific factors contributing to the educational disparity [34].

Second, policy efforts are needed to promote equity in the distribution of factors contributing to educational achievement and attainment. Such efforts may include promoting cognitive and intellectual development among children in OHC, preferably starting at a young age [35]. The differences in school performance and educational attainment, stronger in younger cohorts than the one addressed in the current study [28], are a disturbing reminder that placement in OHC, in which society acts in *loco parentis*, seems to have weak compensatory powers for children from adverse family backgrounds who become ‘wards’ of the state.
